# Reflective practice is a prerequisite for One Health development

**DOI:** 10.1186/s42522-024-00106-8

**Published:** 2024-07-02

**Authors:** Craig Stephen, John Berezowski

**Affiliations:** 1 McEachran Institute, 1859 Delanice Way, Nanoose Bay, Nanoose Bay, BC Canada; 2 Scotland’s Rural College, Inverness Campus, Inverness, IV2 5NA UK

**Keywords:** One Health, Reflective practice, Reflection, Development, Purpose, Critique, Future-ready

## Abstract

One Health is being promoted as a transformative approach in health, conservation, and environmental sustainability. The polycrisis of climate change, pandemics, food insecurity, biodiversity loss, pollution and inequity is creating an urgency to evolve the epistemology and methods of One Health. However, the amount of effort placed into critical and systematic reflection on One Health is outweighed by advocacy for its use, or for expanding its scope of practice. This paper advocates for reflective One Health practice to foster new ways of knowing and doing that are helpful in the face of a rapidly narrowing window of opportunity to preserve the social and environmental factors that secure health and resilience for all species and generations. We propose six areas for reflection; (1) how to moderate conformity so that One Health does not become its own silo; (2) finding the moral purpose of One Health to align actions with desired outcomes; (3) coping with the problem of too many interacting problems; (4) the strategic trajectory of growth to accelerate action on root causes and ensure One Health is future-ready; (5) how to identify priorities across a vast array of problems, values, and needs and (6) how to know if we are making the world healthier and safer and for whom. Reflective practice requires investment in ongoing conversation to guard against over-confidence that we have captured the “one right way” to meet changing expectations and circumstances in a fair and effective way. Our intention is to stimulate thinking and discussion within the One Health community to ensure that “doing is shaped by knowing”. We hope One Health will continue to be an emergent and highly variable set of ever more effective practices that constantly changes in response to the complex, interconnected and changing problems facing the health of people, animals, and the environment.

## Background

Reflective practice involves thinking about what one does and seeing it in a new way [37] by considering types of information and knowledge before, after and amid actions [27]. “It is a necessary hedge against an over-confident belief that we have captured the one universal truth about good practice” [27].

Resources for reflection are rare. In comparison to the many organizations, institutions and publications promoting One Health, published critiques are relatively rare. For example, when we searched PubMed in January 2024, using the terms One Health, criticism, and critique, 230 articles were returned, none of which dealt with One Health. This is not to say that One Health is free of criticism. Inadequate inclusion of the social sciences, inattention to the health of the environment, power imbalances and power grabs, neo-colonial project design, and insufficient attention to knowledge-to-action processes and evaluations are some of the criticisms of One Health [10, 22, 33, 35]. However, the amount of effort placed into critical and systematic reflection on One Health seems significantly outweighed by advocacy for its use, or for expanding its scope of practice. Advocacy for One Health or for the addition of new methods or disciplines without thoughtful, critically reflection risks development of ineffective, inefficient, or even harmful trajectories for the field’s development.

This paper advocates for regular and systematic critical reflection to foster new ways of knowing and doing One Health that are helpful in the face of a rapidly narrowing window of opportunity to preserve the social and environmental factors that secure health and resilience for all. We describe seven themes for collective reflection but do not prescribe response to the themes as response to these themes will be highly context specific.

## Proposed themes for critical reflection

### Moderating conformity

#### Point of reflection: is One Health risking becoming its own *silo*?

Bibliometric studies suggest a tendency for individuals in One Health to associate and interact with other individuals like them [24, 26]. One Health has been called reductionist due to its emphasis on biomedical professionals and perspectives [22]. Most One Health intersectoral action has addressed proximate human health risks and needs, such as zoonotic diseases, with inadequate attention to nonhuman well-being [13, 17]. Medicalization and anthropocentrism have resulted in criticisms that One Health reproduces a western-centric biomedical epistemology, rather than enabling novel intersectoral action, limiting its potential for meaningful change and even potentially contributing to harm [10, 35].

One’s exposure to alternative information and opinions narrows when one only encounters information or opinions that are reinforced by like-minded people. Aligning ideas and information into previously constructed narratives can cement the legitimacy of the narrative, but without consideration of opposing narratives, new perspectives are limited, often leaving problems unsettled [18]. Carducci et al. [7] concluded that researchers who work outside of prevailing narratives or knowledge systems for a field, or who challenge dominant power relations can be marginalized by other researchers, be seen as less legitimate, denied resources, or excluded from a research community. This is borne out by analyses showing that more highly cited interdisciplinary teams most often draw on related knowledge, suggesting a tendency to exclude information that may be too different, groundbreaking or challenging [39]. While this tendency may increase efficiencies and create synergies, it can result in a lack of diversity in approaches and themes considered. Working in teams of like-minded people is comfortable and avoids arguments and criticism but criticism is needed to prevent conformity and group think [9]. As the expectations for One Health grow, so to must critical reflection on how to create teams with sufficient creative capacity and breadth of understanding to design novel and ethical solutions with the greatest chance of success.

### Moral purpose

#### Point of reflection: is why we do what we do aligned with our desired outcomes?

Leading with moral purpose starts with reflecting on why we do something, who (or what) are we helping; how are we making a positive impact; whose values guide what we do; and what is the greater reason for what we do? Finding a single guiding moral purpose is becoming more complicated, as the variety of situations, societies, species, and solutions One Health strives to engage expands.

There is growing recognition that One Health could serve a greater purpose than addressing emerging and pandemic zoonotic diseases. This was reflected in the 2021 description of One Health as an “integrated, unifying approach that aims to sustainably balance and optimize the health of people, animals and ecosystems….. [to] foster well-being and tackle threats to health and ecosystems, while addressing the collective need for clean water, energy and air, safe and nutritious food, taking action on climate change, and contributing to sustainable development” [38]. The challenge with such as expansive scope of practice is not only that the goals can be ambitious and the required environmental and social change hard to achieve but also it risks One Health being a “jack of all trade and master of none.”

Spinoza saw the being of something in what it is not [34]. We should, therefore, ask, what is not One Health? For example, veterinary clinical care and herd health in agriculture dependent communities can “act” like One Health by attending to animal health, reducing public health risks, generating social determinants of human health (ex. income and food security) and reducing environmental impacts without the involvement of cross-sectoral professional teams. As another example, environmental public health has a long history of intersectoral collaboration, many of which already involve veterinary and environmental sectors. Chronic disease epidemiology, health promotion and sustainable development have been working to integrate social and environmental determinants of health well before the advent of One Health. In recognition of the long standing realization that the emphasis in public health on social determinants of health without systematic attempts to neutralize the environmental conditions leading to poor health is increasingly inadequate [1], many fields are working to find ways to create a more holistic approaches to health protection and promotion.

The Quadripartite’s description of One Health [38] is fundamentally an extension of the socio-ecological model of health, which is embodied by the idea of Health in All Policies which can be traced back to the 1978 Alma-Ata Declaration and the 1986 Ottawa Charter for Health Promotion. Health in All Policy is a collaborative approach that integrates health considerations into policymaking across sectors to improve the health of all communities and people. It recognizes that health is created by a multitude of factors beyond healthcare and traditional public health activities. Despite international commitments to Health in All Policy and now to One Health, legal structures and governance frameworks to support cross-policy collaborations are scant and few governments have the political will to implement such comprehensive approaches. Attempts to deliver on the moral purpose of interspecies and intergenerational health equity in One Health- wherein steps taken to protect the health of one species today do not compromise the ability of future generations or other species to meet their own needs-, should be attentive to the implementation gap confronting Health in All Policy approaches.

Pathways to closing the purpose-to-doing gap must be attentive to power struggles and competition for resources that distract from implementing collaborative capacity for change. Time is needed to reflect on; 1) if the aspirational goals are the correct goals, 2) is the direction we are taking One Health going to achieve these goals in the most effective, efficient, and fair way, and 3) how will we know if we are achieving them? Care must be taken not to expand the purpose of One Health simply to gain more profile, authority, or power. We need to ask if working and learning conditions are being transformed in ways that drives growth, commitment, engagement, and change in a right and worthwhile way.

### The problem of too many problems

#### Point of reflection: are we reducing a messy world to a set of interdisciplinary problem?

One Health is being asked to consider different types of health for interacting species, over multiple generations in spaces of different scales that change over time, as do the hazards and harms they experience. Health, as it is experienced in real life, is complex and messy. While there is increased use of the terms systems and complexity in health sciences, “there is some looseness in how they have been translated from their origins in mathematics and physics, which is leading to confusion and error in their application” [29].

No living thing under natural conditions experiences just one problem or one asset at a time. Life is “interprobleminary” wherein various sensitivities, exposures, capacities, and hazards interact, interconnect and compound. Interdisciplinary approaches to a single problem, such as a viral pandemic, does not reflect the interprobleminary nature of life. Techniques such as agent-based modelling are being used to better reflect the complexity of health issues, but they are only as good as their underlying data. Inequities and inconsistencies in effort, capacity, and resources in the three One Health pillars affect data availability, reliability and sharing, leaving us reliant on assumptions about interactions, interrelations, and causation [8, 16]. The predominant research paradigm inadequately considers the interactions between the many factors that can cause a problem to emerge making it difficult to understand the causes of emergent problems, or the effect that mitigations will have on the system and the problems we are trying to solve.

Planning how to deal with complexity begins by understanding which problem for whom one is trying to solve and why a solution is needed. One Health planners need to reflect on if their processes are sufficiently egalitarian and democratic to appropriately characterize the problem and its boundaries. Who, for example, has access to or participates in creating information and knowledge about causes, harms, and the predicted intended and unintended outcomes? Upon deciding on problem boundaries, one then needs to reflect on if they have prepared and assembled the diversity in disciplinary thinking, world views, life experiences, cultural backgrounds, knowledge and value systems needed to work within those boundaries [36]. This requires time, places, and permission to think with others.

### Trajectory of growth and change

#### Point of reflection: what needs to be done to accelerate action on root causes and ensure One Health is future-ready?

At roughly 20 years since the term was coined [12], One Health is still in its infancy. It emerged in response to the need for better knowledge creation and decision making to deal with emerging diseases, primarily of people. It was and continues to be a roughly defined interdisciplinary problem-solving approach combined with the moral purpose of optimizing the health of people, animals, and the environment. Prior to the term being coined, most of what is now called One Health would have been called veterinary public health, infectious disease ecology, or environmental health given that most investment has aimed to prevent, dampen, or respond to human health threats arising from environmental and animal factors.

Investment in One Health has created several benefits (Table 1). While these benefits are helpful, have they been enough to achieve the goals of One Health in our rapidly changing present and highly uncertain future? Many areas of study and policy struggle to translate evidence at rates and scales sufficient to inspire and sustain actions against interwoven health threats felt across spatial, species and temporal boundaries. One Health is now being promoted at national and international fora as a transformative approach in health, conservation, and environmental sustainability, implying an expectation to evolve beyond its historic focus on zoonotic diseases. But, has One Health been evolving for a world that is disappearing rather than for the world that is coming—a world with less standardization and predictability but heightened connectivity and polarization; a world with shifting demographics and power dynamics; and with unprecedented environmental change? Ways of thinking and doing need to evolve to help decision makers, policy makers and researchers deal with an uncertain, volatile, complex, and ambiguous future. “Business as usual” based on past concepts of success and progress may no longer be a viable pathway.

**Table 1:** Benefits arising from past One Health initiatives

	**Benefit**
Intelligence	Decision makers can have a broader sense of the risk landscape and act earlier with greater confidence on strategically selected intervention targets because of broadened sources of information used to anticipate and assess threats and open a larger solution space.
Knowledge transfer	Knowledge sharing between sectors helps transfer experiences, information, and expert insights, exposing decision makers to a broader understanding of the problem they face and new ways of conceiving and managing a problem, thus reducing the chance of undesirable consequences
Co-benefits	By collaboratively working throughout the causal chain of a complex health problem, co-benefits can occur that have short- and long-term effects, creating co-benefits to collaborators, thus encouraging ongoing collaborations.
Efficiency of resource use	Coordinated and synergized collaborations that allow partners to apply their skills, knowledge, and resources to aspects of a shared problem can reduce resource waste and unintended consequences that delay progress on health goals.

Knowledge about threats and risk factors alone will not lead to risk reduction without understanding the factors changing the trajectory of a socio-ecological system to a safer state. Without revolutionary change to address root causes of health and resilience rapidly, effectively, and collectively we will continue to battle new crises as they emerge. The polycrisis of climate change, pandemics, food insecurity, biodiversity loss, pollution pressures and inequity amplifies the need for interspecies and intergenerational health equity [32]. Research and learning that break traditional barriers and that are action oriented are urgently needed [3, 5].

Integrating traditionally distinct knowledge, skills and perspectives for disruptive purposes is a growing expectation of universities, businesses, and governments [19]. Disruption comes from introducing new ideas and approaches, asking fundamental questions that break the status quo, and pushing inquiry in new directions [6] while at the same time cultivating future-ready skills, knowledge, and attitudes. Disruptive One Health must be more than just integrating human and animal healthcare systems for better disease control.

Revolutionary changes won’t occur if we drive innovation through old ways of knowing and doing. When education, funding and evaluation emphasize the mastery of facts and skills, we are drawn away from developing game changing solutions [11]. Revolution requires reflection on the social norms, beliefs and values underpinning One Health and on the necessary attributes and competencies of those charged with inspiring and leading change. It also requires reflection on the changes we want to make.

### How are priorities set?

#### Point of reflection: how do we decide priorities across such as vast array of problems, values, and needs?

It is naïve to assume that human, animal, and environmental health programs have equal power, profile, resources, or capacity or that they are treated equitably. For example, killing millions of animals and accepting the resulting animal welfare and waste disposal implications to reduce zoonotic diseases risks to people or the impact on economies arguably does not treat animals’, peoples’ and ecosystems’ health equitably. Even within a sector, power dynamics and social risk perceptions influence which issues are the focus of action and investment and which remain unaddressed. In an ideal One Health world all options for prioritizing and addressing a problem would be considered based on the best available evidence and would equitably maximize the health of all species and generations occupying a shared space. However, arguments about the severity and imminence of an issue are always filtered through power and perception [4]. The imbalance between interest and investment in viral zoonoses versus pollution versus climate change in One Health is an example. The entanglements of power and participation in One Health can be many and influential [28] but empirical explorations of the impacts of power dynamics in transdisciplinary undertakings are limited [15].

Not only can power dynamics influence the fairness and effectiveness of a team’s effort, disproportionate power of select groups advising organizations and agencies that establish funding and policy priorities can have national and international implications. Increased attention to One Health is cultivating power struggles between human and animal health stakeholders and increased funding has created the potential for more power struggles by expanding the number and variety of stakeholders who shift in directions most aligned with their own interests, potentially splintering and weakening the movement [25, 31].

Being able to direct or influence the behaviour of others provides power to control agendas, frame problems, promote methods or ideological trajectories, establish governance systems and tell the resulting stories of success or failure [2]. To better understand how prevailing narratives are setting priorities and how those priorities are addressing the problems for whom, one must reflect on who initiates One Health undertakings, for what purpose, who participates, and how they participate. Such reflections have pragmatic implications. For example, a narrative that emphasizes eliminating a problem or risk can cause the global community to focus on a single, definable response to a forecasted threat rather than sustaining focus on the suite of circumstances that build general capacity to resist or mitigate harms arising from wicked problems like biodiversity loss and climate change. Reflection on the ethical foundation of One Health is needed to guide decision-making that accounts for power, responsibilities, dependencies, and obligations.

### How does it work?

#### Point of reflection: do we know that One Health makes the world healthier and safer?

A transformative agenda needs to move beyond discovering what works and why, to what works for whom and under what circumstances. In an increasingly dynamic, complex, and nonlinear world there is a growing need to actively engage with, continuously reflect on, and adapt to changing circumstances [21]. It is no longer enough to try to document and explain things; one also must try to change them and be continuously involved in the processes of change. Yet a recent review suggested that a minority of One Health literature is focused on action [17].

Investment in creating evidence-based strategies is too often not prioritized, especially when resources are limited and there is an urgency to act. There is sparse empirical evidence of effectiveness or comparative impact of intersectoral approaches to health despite the strong belief that they are essential to remedy significant public health problems [23]. Concerns have been raised about how the inappropriate use of mixed methods research can create false confidence in their capacity to solve fundamentally unsolvable problems [14]. This is not to say that the collaborative methods and perspectives that underpin the One Health concept are not useful, reliable, or repeatable. Rather, it is to ask us to reflect on whether we have been evaluating the right things the right way.

One Health evaluations have tended to focus on issues such as cost savings, improved data flow, expanded knowledge bases, or changes in a risk behaviour. Systematic comparison to counterfactual situations, examinations of unintended consequences, and determination of what attributes of a program result in sustainable improvements in health outcomes are rare. This is, in part, due to the lack of funding for One Health implementation science. There are also unresolved challenges in assessing cause-effect relationships in complex dynamic systems both to understand the causes of problems and how solutions will change complex systems.

The diverse situations, scales, epistemologies, values, and specialities that exist across the scope of One Health practice makes it exceedingly hard to answer the questions of whether it works and it has been helpful. An effective intervention in some settings could be ineffective or harmful elsewhere. Determining what caused what can be extremely challenging when a problem is influenced by multiple components with multiple independent or interdependent interactions acting across blurry boundaries– a dilemma faced by many fields attempting to understand and manage health holistically.

Defining success through a mechanistic, biomedical framework or in economic terms alone rather than considering a broader social and ecological context will create an imbalance in how success is perceived, and impacts achieved. To evoke One Health as a solution without specification of the precise multidimensional problem to be solved is akin to selecting a method before developing a hypothesis.

## Conclusion

Finding time and space to reflect is essential in our highly networked, complex, and dynamic times [20]. Systematic, rigorous, disciplined thinking with others helps to move to a deeper understanding of the meaning of something and its connections to other experiences and ideas [30]. It requires ongoing conversation about present action, past experience, intentions for the future, and the willingness to revise our own truths [34].

One Health has not yet developed into a unified set of successful problem-solving methods. Rather it has developed in many directions. In some cases, the term One Health has been adopted as a label for well known methods used under pre-existing labels. In others, methods from other disciplines such as the field of transdisciplinary science have been added to the One Health toolbox. Because of its infancy, we can expect both One Health methods of practice and the epistemology of One Health to continue to emerge on multiple fronts. This is not a bad thing. Creating many approaches increases the likelihood that at least some of them and their outcomes will be useful. Effective rapid sorting of the approaches into useful and non-useful categories is essential for rapid advancement. Evidence is essential.

However, due to the challenge of interproblemarity, monitoring the impact on single outcomes (e.g., disease, death, change in abundance of a single species in the present time) can lead to biased estimates that inadequately consider impacts on other species, the environment or future residents. Systematic, rigorous, disciplined reflection by a wide range of people is essential for more rapid and effective improvement in One Health policy and practice. There is an urgency to improve One Health practice because of accelerating climate change and the yet unknown effects it will have. We have provided some themes relevant to One Health that we consider important for reflection. We have not provided answers, as we are hoping that these themes will inspire One Health practitioners and epistemologists to apply them to their own situations. We are hoping to stimulate thinking and discussion within the One Health community that will result in the emergence of more themes for reflection, and that these will be published and used by the community to improve the effectiveness of One Health practice more rapidly. We hope One Health will continue to be an emergent and highly variable set of ever more effective practices that constantly changes in response to the complex changing problems facing the health of people, animals and the environment on this planet.

## Data Availability

Not applicable.
